# Crisis in care homes: the dentists don’t come

**DOI:** 10.1038/s41405-021-00075-4

**Published:** 2021-06-08

**Authors:** Rakhee Patel, Mamoona Mian, Claire Robertson, Nigel B. Pitts, Jennifer E. Gallagher

**Affiliations:** 1 King’s College London, Faculty of Dentistry, Oral & Craniofacial Sciences, Centre for Host Microbiome Interactions, Denmark Hill Campus, London, UK; 2 King’s College London, Faculty of Dentistry, Oral & Craniofacial Sciences, Dental Innovation and Translation Hub, Guys Hospital Campus, London, UK

**Keywords:** Gerodontics, Dental public health

## Abstract

**Aim/Objectives:**

To present the findings of the challenges relating to access to dental care for older people in care homes from the Fluoride Interventions in Care Homes (FInCH) Trial.

**Methods:**

Thematic analysis of 11 interviews / focus groups with care home managers and care staff were carried out against a framework informed by the literature drawing on lived experiences.

**Results:**

The challenges identified mapped to Penchanksy and Thomas’s (1981) five dimensions of access but also highlighted themes specifically relevant to the care home population. These include a lack of suitable services for routine and urgent domiciliary and clinic care, complex referral processes, operational challenges in the need for appropriate care chaperones, expectations of information for dental charge exemption and capacity / consent processes within the home.

**Discussion:**

There is a malalignment of dental services offered to meet the needs of care home residents which has resulted in a reactive dental care system that is not fit for purpose and an entire generation of older people living in care with dental neglect.

**Conclusion:**

Urgent action is needed to commission not only the appropriate quantities of both routine and urgent dental care, but ensure it is delivered by clinicians who are appropriately skilled to meet the high levels of dental needs in an increasingly medically and behaviourally complex care home population. In line with eye tests and prescribing at the very least, free routine dental examinations should be offered to all care home residents, creating the opportunity for advice and prevention, and enabling care home residents to function and be free of pain.

**Trial Registration:**

The FinCH Trial registration EudraCT number 2017-002248-34.

## Introduction

It is well known that the UK population is ageing, and this section of the population is growing at a faster rate than any other age group. In 1999, around one in six people were aged 65 years and over (15.8%). This increased to one in every five people in 2019 (18.5%) and is projected to reach around one in every four people (23.9%) by 2039.^1^ This growth represents an additional 7.5 million people aged 65 years and over in the United Kingdom (UK) in 50 years’ time. It is important that we support them to sustain their oral health through older age to their end of life.^2^

The COVID 19 pandemic^3^ has shone a light on care homes in England^4^ and across the UK but the problem of a neglected and severely underfunded social care system has been long standing. We now have a complex social care landscape, with a mixed economy which is straining under the pressure of an ageing population with increasing care needs.

Change has dogged the care home sector over the past two decades in relation to health and social care. The 2009 Personal Care at Home bill empowered and enabled people with care needs to stay at home for longer by providing ‘at home’ care. This has resulted in people entering care homes later in life and with high levels of care needs. The 2016 Report by Laing and Buisson estimated approximately 4% of people aged over 65 years lived in care, rising to 15% of those aged 85 or more.^5^

In parallel to the above, there was also change in dental care delivery for care homes. The National Health Service [NHSE] dental contract for England introduced in 2006 meant that general dental practitioners were no longer able to provide, and claim for, domiciliary care as part of the routine dental contract, thus immediately limiting access to dental treatment for care homes. A small number of domiciliary contracts were retained, but these were not at specialist level and located on the basis of history.

In addition, as well as having increased personal and medical care needs, and limited access, the dental needs of older people have also changed over this period. People are retaining their teeth for longer, with the 2009 Adult Dental Health Survey showing 53% of adults over the age of 85 years had retained some of their dentition, with an average of 14 teeth.^6^ A greater proportion of older people with natural teeth, limited self-care abilities and increased vulnerabilities mean care home residents are at significantly higher risk of dental diseases. Evidence shows significant differences between institutionalised and community dwelling older people, with those in care having fewer teeth and significantly higher levels of dental decay.^7,8^ Gerodontology, as an area of dentistry which deals with the diagnosis, management, and treatment of dental conditions relating to older people, is a slowly emerging area of expertise. But there are current challenges with the absence of an appropriately sized, trained and experienced workforce to deliver the level of specialist care needed for these complex patients across the whole dental care system.^9^

More people living in care homes, with higher levels of personal care and dental needs, the challenges around the Gerodontology workforce, and inadequate funding resulting in a model of reactive dental care where there is limited scope for regular care and prevention,^10^ means we now have an older, vulnerable population in care homes suffering from dental neglect.

The emergence of the oral health challenges in care homes is highlighted and evidenced by several policy and guidance documents by the National Institute of Clinical Excellence,^11^ the Care Quality Commission,^12^ and Public Health England.^13^

In order to understand more about tackling the issue of poor oral health in care homes funding was secured from NIHR for a feasibility study. The Fluoride Interventions in Care Homes (FInCH) trial is a randomised controlled assessor blind parallel group (3 groups) trial with the aim of establishing the feasibility of delivering fluoride interventions to prevent dental decay in care homes (Trial registration EudraCT number 2017-002248-34).

The primary objective of the overall study is to establish the feasibility, practicability and compliance of fluoride interventions to prevent dental decay in care homes, whilst the secondary objectives include exploring the acceptability of the interventions from resident, care home and dental services perspectives, and the impact on of the interventions on resident’s quality of life.

## Aim

The purpose of this paper is to report on some of the qualitative findings from the NIHR funded Fluoride Interventions in Care Homes (FInCH) Trial, in relation to the challenges and issues of access to dental care for older people in care homes, from the perspectives of care home managers and care staff.

## Methods

The feasibility trial was conducted in one outer London borough, selected as it has some of the highest numbers of care homes for older people in the city which forms a region of England. To be eligible to participate, care homes needed to be for the care of the elderly (>65 years), have between 60-80 beds, and have passed council inspection. Of those eligible, a convenience sample of three residential and three nursing homes were approached.

As part of the nested qualitative component of the study, interviews with care home managers and care home teams were conducted across six homes to explore their lived experience of caring for older people. The profile of each care home and participating team member is presented in Table 1.

**Table 1:** Information on care homes and interviewee participants in the FInCH Trial.

Care home identifier (CH)	Care home type	Number of beds	Method of qualitative data collection	Participants	Gender
CH1	Residential	70	Interview	Manager and Deputy Manager	Female and Female
CH2	Residential	68	Interview	Manager	Male
			Interview	Carer	Female
CH3	Nursing	56	Interview	Manager and Deputy Manager	Female and Female
CH4	Nursing	54	Interview	Manager	Male
			Interview	Deputy Manager and Lead Nurse	Female
			Focus Group	Carers (3)	Mixed Genders
CH5	Nursing	60	Interview	Manager	Female
			Focus Group	Carers (4)	Mixed Genders
CH6	Residential	84	Interview	Manager	Female
			Focus Group	Carer (4)	Mixed Genders

Ethical approval was granted (IRAS 202190) prior to study recruitment commencing by an NHS research and social care ethics committee. Care home managers and staff were invited to participate by means of an invitation email/letter and information sheet. All participants provided written informed consent prior to participating in the interviews, and the study team ensured that all aspects of the Declaration of Helsinki were met prior to commencement of interview.

Interviews and focus groups were conducted by a single trained interviewer (RP), with the use of a topic guide informed by the literature.^14^ The topic guide contained a series of open questions, with the purpose of generating an in-depth understanding of the type and extent of barriers, as well the possible solutions for delivering personal mouthcare and accessing dental care, drawing on their lived experiences.^14^

Interviews and focus group discussions were audiotaped, and transcribed verbatim by a transcription service under a confidentiality agreement.

A rigorous matrix-based approach to qualitative data management and analysis was adopted as outlined by Spencer et al. (2013).^15^ This was undertaken in several stages by MM and RP working with JEG. First, a literature review was conducted on access to dental care in UK care homes drawing on Penchansky and Thomas’s (1981) theory of access to care,^16^ whereby search terms around availability, acceptability, accessibility, affordability, and accommodation were included. A total of 473 articles were published in the previous five years were identified, out of which 39 addressed the oral health of people living in care homes and 11 addressed their access to professional care. Analysis of this literature provided an initial framework of themes and sub-themes, which as well as informing the interview topic guide, provided the basis for analysis of the data.^15^

Second, transcripts were read and re-read for familiarisation and to identify the themes from the initial framework and any new themes within the data; the framework was adapted accordingly.^15^ Third, the framework of was then applied to the data within Microsoft Excel where the data were then coded/indexed and then sorted by theme and sub-theme, and reviewed.^15^

The data were summarised and categorised by themes across the transcripts,^15^ whilst being mindful to summarise in way that the context and essence of each participant’s view was not lost by grounding the analysis in the data, keeping useful expressions and phrases and using direct quotations.

Finally, a description of the findings and explanation of the patterns in the data was developed.^15^ We will focus on the explicit accounts provided by the participants as well their perspectives on social care policy and planning.

## Results

**Fig. 1:**
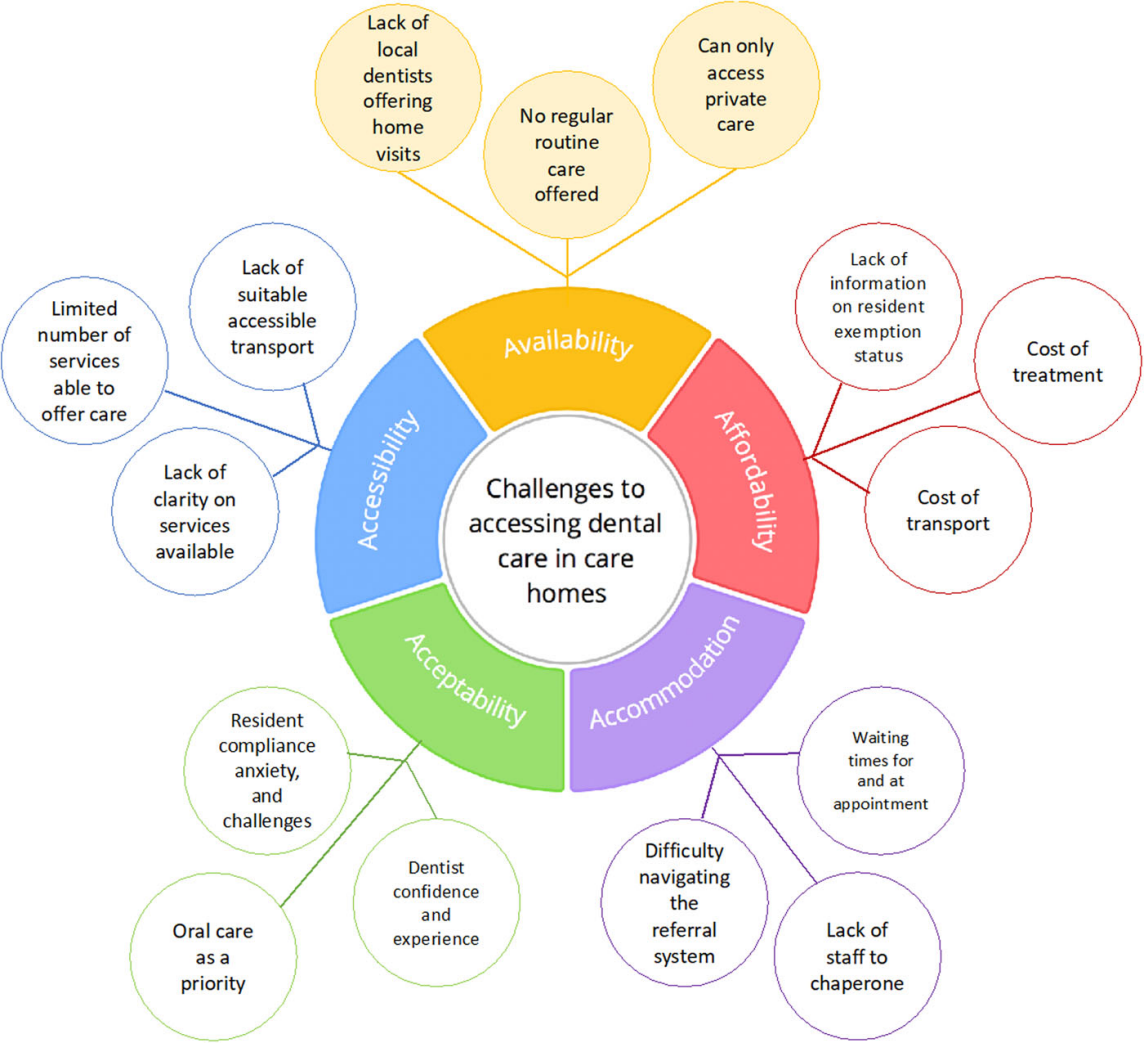
Image depicting the challanges to accessing dental care for care home residents mapped to Penchansky and Thomas’s theory of access to care (1981) domains. These include challanges of individual and organisation priorities, barriers and facilitators as well power.

Qualitative analysis of the interviews and focus groups with care managers and care staff identified access to dental care themes including the individual and organisational priorities, barriers and facilitators and power. These themes are summarised in Fig. 1.

Access to care related to individual’s philosophy of oral health and perception of need, and the organisational priorities. It was accepted by care staff that dental care was accessed only when a problem arose, *‘…most of them when they come to the care home, they mostly see the dentist when they need to.’ (Participant 19)*. Oral health was a low priority for residents, as ‘*… some of them [residents] just can’t be bothered (Participant 15)’*. On deeper exploration, carers’ own beliefs about ageing and oral health played a significant impact, as demonstrated in the statement from a manager *‘I think people[carers’] just tend to see… oh old people – no need for dentist – and that’s just pushed to the side.’ (Participant 15)*.

These beliefs intertwined with the reactive approach to seeking access to care were heightened by the challenges of delivering oral care, especially for those with behavioural challenges such as dementia. Presence of dementia was mentioned to be a significant challenge in ascertaining the need for professional dental care. The importance of regular domiciliary dental contacts with the same people/person was highlighted ‘…*regular dentists would visit and then it would help, and I think with the residents living with dementia if that consistency is there as well it becomes, okay…. So that’s become a routine for them and so there’s no issues getting their toenails cut and all that so’ (Participant 15)*.

Widespread challenges in finding a dental practitioner, NHS or privately were raised. The frustration of the dichotomy of guidance and reality was clear whereby, ‘*If we are saying oral care is so important then there needs to be services that are available’ (Participant 1)*.

Although there was a preference for domiciliary care, ‘*we just need an NHS dentist that’s happy to come and see residents in the home’ (Participant 1*). There was an understanding of the challenges and limitations of domiciliary care ‘*it’s very difficult for the dentist to come here and when they do come you know, in a home, they’re kind of limited in what they can do’ (Participant 19)*.

Managers were keen to build local relationships with dental services, ‘*If we can establish a point of contact because we’ve been trying to establish a contact with a certain dental practice who can come and visit the home at least, then that would be helpful for us’ (Participant 14)*. In almost all interviews, there was a feeling of frustration that dental practitioners were unwilling to visit the homes *‘Unfortunately, none of the dentists would like to come and visit because obviously CQC, they said guidelines, they need to check with the equipment that they need to use and so forth and so on, unfortunately that is something we always hear’ (Participant 14)*. Participating managers reported turning to other services for support in the absence of a dental provider ‘*if we’ve got a resident with toothache and we don’t have a dentist, we’ll just go to the GP’ (Participant 1)*.

When available services were identified, other aspects of accessibility became a significant challenge. This was discussed at several levels. Lengthy referral processes were deemed burdensome, requiring significant time and additional staff, specifically nurses, to fill lengthy online referral forms; ‘*it was a whole performance to get the referrals’ (Participant 1)*. Once residents are referred into care, managers reported longer wait times for dental visits than other healthcare services, *‘So, it’s like going back and forth in a circle and the poor resident in the end, some of them never got the treatment they needed. Some of them ended up with, em, passing on, God bless their soul, without getting any treatment’ (Participant 13)*. There was a feeling of a lack of joined up coordinated care for residents, and no consistent pathway that could be accessed, *‘We want to be able to be confident that we’ve got a port of call… Just to let that resident know. If it is a real emergency – here’s somebody else, you can contact’ (Participant 1)*.

A significant barrier to care, and reason for the preference for domiciliary care, was the lack of suitable services for wheelchairs ‘*it’d [had] got steps [into the dental practice] or things like that, that’s, that’s the barrier for us, that’s when there’s a problem, because not all dentists, you can just, wheel your wheelchair straight in, um and that was that problem’ (Participant 22)*. The lack of suitable transport was also highlighted, ‘*because lots of them have got mobility issues and then there’s a few taxis that carry people with wheelchairs. You need to transfer them from the wheelchair onto the chair and most of them won’t do it. So that’s one of the challenges’ (Participant 15)*. Cost of transportation was also a significant issue, *‘If they haven’t got money to pay for the dentist how are they going to have money to pay for their transport? So, who is then going to fund that transport…The ones who are local authority that are bed bound that can’t leave the home or if they can they need to go stretcher by transport, that don’t have any money they are the ones that struggle when they got a toothache…if they do need treatment to have somewhere to go, where transport is then arranged for them to go and have their dental health care. Especially if they are residents that have no money. They are the ones we worry about, the ones who have no money are the ones that are left behind’ (Participant 1)*.

Cost was a recurrent theme throughout multiple aspects of the interviews relating to both NHS and private care. There were significant disparities in access to care between those who were able to fund the care themselves, and those that were seeking funds from family, or that were possibly exempt. *‘I think the relatives would want to be free to know, look, I can call them now, and look, your mother needs to go to the dentist, and if it’s free, they will say yes straightaway, but it’s oh you need to pay’ (Participant 21)*. There was a clear lack of understanding of dental charge exemptions; ‘*who’s entitled to have free dental treatment, I wouldn’t know, I don’t know how it works actually’ (Participant 13)*.

In one home the inability for the home to determine if the resident is exempt from dental charges was highlighted ‘*That’s the thing and I don’t know how, [or] where you could find that information. Because normally we just go out and assess, they come in, or they’re referred, let’s say, with [by] the local authority, but we don’t have that information – if they were on any benefits. So, I think that’s where the problem starts’ (Participant 13)*. This was magnified by the fear of penalty ‘*if you fill this form out ‘wrong’ you are liable for charges. It makes you feel like oh my gosh I don’t want to fill this form out because I am only filling it out from the information that I do have but if I am wrong then I can be in trouble or giving the wrong information even though there’s nowhere for me to collect the information from’ (Participant 1)*.

For some managers, accessing existing funds through next of kin was an issue, ‘*but it’s sourcing the next of kins, the next of kins have just disappeared…But then, they’ve got money, but the family has just disappeared. They probably will turn up when they’re dead, but then… which is sad’ (Participant 15)*.

Managers advocated that dental treatment for older residents should be free of charge and raised the malalignment of the policy narrative to other healthcare services offered ‘*I think it should be something that is part and parcel of the NHS and of residents in a nursing home. And we understand obviously how expensive that can be but if we try to say, actually - oral care is just as important as eyesight, why is there a difference?’ (Participant 1)*.

The themes and sub-themes were underpinned by the sense that the management and their staff had responsibility for vulnerable residents, but little power. This was reflected in the fact that when they perceived needs of the resident, they were not able to get the family or system to act in a timely way or at all to make appropriate arrangements.

**Fig. 2:**
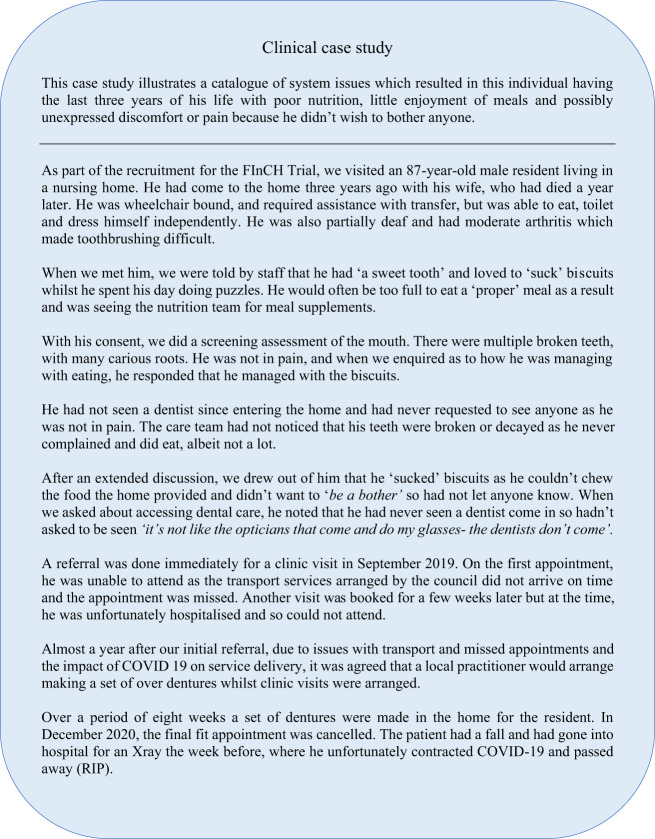
Clinical case study exemplifying the systematic challages faced in accessing care.

Furthermore, the case study presented (Fig. 2) provide insight to one resident’s needs and the systemic challenges faced in accessing care.

## Discussion

The research we have conducted highlights the challenges in accessing dental care for dentate older people in care homes who have needs that are not being met. We are not only seriously failing our most vulnerable older adults and but also causing social care staff dilemmas in care delivery. These challenges map to Penchanksy and Thomas’s (1981)^12^ dimensions of access but also highlight themes that specifically relevant to this distinctive population. The lack of suitable services and experienced clinicians, compounded by the need for appropriate care chaperones, lack of exemption information and no routine care services, has resulted in a dental care system that is not fit for purpose and an entire generation of older people in care with dental neglect.

The findings of this work support the existing literature,^11–13^ highlighting the unmet dental needs of residents in care homes. It would appear that because of the commitment to keep people in their own homes for as long as possible, only those with a high level of care needs are entering care homes. These needs may be behavioural, physical, psychological, medical or a combination of these. For many, by the time they enter care, their ability to care for themselves has significantly declined, and they require attention and care on all fronts. In line with a high level of general care needs, these individuals also have significant dental needs, including pain, sepsis, a non-functional dentition, poorly functioning prostheses and tragic impacts on diet, nutrition, speech and quality of life as exemplified by the case study presented.

The cost of dental care, and processes associated with cost, present a huge barrier for care homes, their residents and families. The mixed economy of care home funding means inequalities between care home residents exist, whereby those who are mobile, able to pay for transport and dental care may be able to access dentistry, but residents that require specialist transport, or who have little disposable income through their own means or family, are often reluctant to access dental care as they are unsure of payment.

This is compounded by a mismatch between dental exemption criteria and social care funding. When a person requires a care setting, they are means assessed for local authority support or if they have medical needs, may be NHS funded. The care home will not have any financial record of the benefits the resident received prior to entering care; they will only be informed of the funding arrangement for the care home stay. As a result, they cannot verify or evidence whether a resident meets the exemption criteria for dental care. The fear of penalty and need for legal power of attorney’s involvement and consent, often results in delays in accessing care. The policy and dental payment system are misaligned with that of social care which presents a major and unnecessary barrier to care homes and residents, creating inequalities to access between and within homes.

Urgent action is needed to enable social care to prioritise dental care in the same way they view access to pharmacy, optometry and medical services. Free dental check-ups at the very least, should be considered to overcome some of the barriers associated with exemption and cost, and align dental care with other healthcare services, such as free eye tests and prescriptions for people over the age of 60 years. This would also create the opportunity for advice and support to the care home staff, resident and family, and a mechanism for ongoing support of the preventive regime. This continual point of contact is vital in supporting and changing the culture and attitudes to oral health in the population living in care homes which is a evidenced barrier.

Clearly the current out of date system requires considerable immediate transformation including a review of funding, workforce and resources. A longer-term restructuring of dental care services to meet the needs of our ageing population is required. There are several challenges and barriers that need to be overcome to deliver a comprehensive care pathway for care home residents that is person centred and integrated within dental care services and the wider health and social care system. It is important we consider opportunities for reformation across the system for long term sustainable change.^17^ Services should be co-developed in partnership with social care experts, so that there is a shared understanding of the challenges from both perspectives and opportunities to explore novel and useful solutions are sought.

Although the events of the last year have been devastating for the social care landscape, they have demonstrated opportunities in embracing the use of technology. During the COVID pandemic care homes have been forced to adapt to using virtual technologies for almost all their training and health care needs, including medical care which now offers an opportunity for tele-dentistry in a way not possible previously. This should be explored and funded to assess if this as a feasible adjunct to face to face visitation to overcome challenges around transportation and patient compliance in a cost-effective way.

The key to change for care homes is to fund not only the appropriate amount of routine and urgent dental care, but develop an appropriately sized and suitably skilled workforce who can deliver high quality and appropriate dental care for a patient base with high levels of dental need, complex medical histories, multiple behavioural challenges and mobility issues. This expertise currently lies in our community dental services and specialist services. In the immediate term, these services need to be funded appropriately to enable coordinated, well communicated routine and urgent care services designed to meet the needs and complexities of the large number of people in care homes and the care home landscape.

In the longer term there needs to be increased funding to expert and specialist services, restructuring of general dental services alongside upskilling of the dental workforce in managing disease and pragmatic treatment planning for these complex patients. This should be done concurrently to ensure a coordinated approach for care home residents to see the right person, with the right skills for the right care to meet their individual needs and wants.

## Conclusions

In conclusion, the findings from this research highlight the urgent action required to commission the appropriate quantity and levels of expert services necessary to meet the high levels of dental need of an increasingly complex care home population. Care home residents deserve fair and equitable access to routine and urgent dental care to enable them to maintain function and be free of pain. Significant barriers for care homes and residents are the issues related to payment and exemption. Free dental check-ups, at the very least, for all care home residents should be considered.

Furthermore, upstream approaches in creating coordinated, patient centred, integrated care pathways for people as they age are equally important to prevent future generations ageing and entering care with deteriorating dentitions and depleting oral health related quality of life. This should be done in partnership with social care to understand their processes and challenges so that any commissioned service is sustainable and is fit for the purpose of meeting the needs of the individual and the system.

## Data Availability

The datasets used and analysed during the current study are available from the corresponding author on reasonable request.
